# Seedling and adult plant resistance to leaf rust in some Bulgarian common wheat lines

**DOI:** 10.18699/VJGB-23-54

**Published:** 2023-09

**Authors:** V. Ivanova

**Affiliations:** Agricultural Academy, Dobrudzha Agricultural Institute, General Toshevo, Republic of Bulgaria

**Keywords:** wheat, Puccinia triticina, pathotypes, adult and juvenile resistance, пшеница, Puccinia triticina, патотипы, ювенильная и возрастная устойчивость

## Abstract

The response of 250 common winter wheat breeding lines was investigated for resistance to the causative agent of Puccinia triticina under conditions of an infected field on the territory of Dobrudzha Agricultural Institute – General Toshevo, Bulgaria, during three successive seasons. Twenty lines with different degrees of resistance under field conditions were selected. Multi-pathotype testing was used to study the response of these lines at seedling stage under greenhouse conditions to individual pathotypes of P. triticina. Based on the response of the lines at seedling and adult stages, we found out that 20 % of them carried race-specific resistance. One of the lines (99/08-52) reacted with full resistance to the pathotypes used under greenhouse conditions. The reaction demonstrated by this line coincided with the response of isogenic lines carrying the genes Lr9, Lr19, Lr22a, Lr22b and Lr25. The other three lines (19/06- 108, 82/08-43 and 82/08-35) showed a resistant reaction to 6 or 5 of the pathotypes used in the study. Their response partially coincided with the reaction of 5 isogenic lines, and the presence of some of these genes in the above lines is quite possible. Lines carrying this type of resistance are to be subjected to further genetic and breeding investigations to prove the presence of a race-specific gene. Twenty-five percent of the lines combined partial race-specific resistance at seedling stage with the resistance of race non-specific nature at adult stage. Forty percent of all studied lines carried race non-specific resistance, and 15 % of the lines possessed resistance of the “slow rusting” type. As a result of the study we carried out, the lines that demonstrated stable resistance to leaf rust can provide sufficient protection of the host and can be included in the breeding programs for developing varieties resistant to P. triticina.

## Abstract (ru)

Проверена реакция 250 селекционных линий мягкой озимой пшеницы на устойчивость к возбудителю Puccinia triticina в условиях инфекционного поля на территории Добруджанского сельскохозяйственного института (Генерал Тошево, Болгария) в течение трех сезонов. Отобрано 20 линий с разной степенью устойчивости в полевых условиях. Для изучения реакции этих линий на стадии проростков в тепличных условиях на отдельные патотипы Р. triticina использовали мультипатотипное тестирование. По реакции линий на стадии проростков и взрослых растений установлено, что 20 % из них обладают расоспецифической устойчивостью. Одна из линий (99/08-52) проявила полную устойчивость к патотипам, используемым в тепличных условиях. Ее реакция совпала с реакцией изогенных линий – носителей генов Lr9, Lr19, Lr22a, Lr22b и Lr25. Еще три линии (19/06-108, 82/08-43 и 82/08-35) показали резистентную реакцию на шесть или пять патотипов, использованных в исследовании. Их реакция частично совпала с реакцией пяти изогенных линий. Вероятно, некоторые из этих генов присутствуют у перечисленных выше линий. Линии-носители данного типа устойчивости подлежат дальнейшим генетико-селекционным исследованиям для подтверждения наличия расоспецифического гена. У 25 % линий частичная расоспецифическая устойчивость на стадии проростков сочеталась с устойчивостью расовой неспецифической природы на взрослой стадии. Из всех изученных линий 40 % обладали расовой неспецифической устойчивостью, а 15 % – устойчивостью типа slow rusting. По результатам наших исследований, линии, показавшие стабильную устойчивость к бурой ржавчине, могут быть включены в селекционные программы по созданию сортов, устойчивых к Р. triticina

## Introduction

Leaf rust is one of the most widespread diseases on wheat in Bulgaria and one of the most important diseases in those parts of the world, where wheat is the main cereal crop. The development and growing of resistant cultivars is an important, efficient, environmentally friendly and cost-effective method for control of the disease (Bariana, 2003; Bariana et al., 2007; Singh et al., 2016; Volkova et al., 2020; Kokhmetova et al., 2021). In order to avoid the danger of epiphytotic occurrence, it is necessary to have at our disposal a large number of sources – carriers of different genes or types of resistance, which should be properly alternated in the production fields (Donchev et al., 1977). According to Van der Plank (1963), the resistance can be categorized into two classes based on the genetic control and the phenotypic effect – race specific (vertical) and race non-specific (horizontal).

The specific resistance is determined by one or several genes acting independently of one another and is efficient to individual races of the pathogen (Roelfs et al., 1992). Each gene ensures resistance to all races that do not have a respective gene for virulence, but not to races that do not possess such a gene. When this resistance is realized in a widely distributed cultivar, high selection pressure on the pathogen occurs, leading to the formation of new races with new genes for virulence, i. e. this type of resistance quickly loses its efficiency, because the pathogen population evolves (Huerta-Espino et al., 2011, 2014; Lowe et al., 2011; Ellis et al., 2014).

The non-specific (horizontal) resistance ensures protection of the plants against all races of the pathogen and the genes, which determine it, have additive effect. The polygenic nature of this type of resistance is the reason for its durability (Parlevliet, Zadoks, 1977; Singh et al., 2011). It is expressed at adult stage and its mechanism consists in reduction in the amount and rate of the disease (Stubbs et al., 1986; Li et al., 2014). The impact of the qualitative resistance of the host on the evolution of the pathogen populations is less documented in the literature (Volkova et al., 2020). It has been shown that the fungal pathogens may evolve and adapt to qualitative resistance through breeding for higher aggressiveness (Delmas et al., 2016; Frézal et al., 2018).

Especially interesting is the resistance of a non-specific nature – the “slow rusting” type, or the retarded development of the pathogen. The cultivars possessing this type of resistance allow the pathogen to sporulate on them, to attack them to a moderate degree, without forcing the pathogen to develop new more aggressive races (Knott, 1989; Kolmer, 2013; Singh et al., 2016). The genes determining this type of resistance are related to such factors as pustule size, infection frequency, latent period, and are most often defined as “slow rusting genes” (Caldwell, 1968; Kolmer, 1996; Ellis at al., 2014). Although the genes determining adult stage resistance are considered to determine durable resistance, some authors point out that the occurrence of new and aggressive races of the pathogen may make these genes inefficient (Singh, Rajaram, 1992; Park, McIntosh, 1994; Huerta-Espino, Singh, 1996). This is the reason why it is necessary to search for and develop new sources of resistance (Pathan, Park, 2006; Ivanova, 2015; Ivanova, Chamurliisky, 2017; Ivanova et al., 2019a, b). Hussain et al. (1999) concluded that durable rust resistance mechanism in wheat is achieved through incorporation of partially resistant minor genes, which seems to be more appropriate for sustainable wheat production.

The method based on the “gene for gene” relation is one of the fundamental concepts of the relationship between the plants and the pathogens (Flor, 1956). Based on this hypothesis, Person (1959) developed a method for identification of genes for resistance with the help of testing races of the pathogen, which carry certain virulence. The multipathotype test used for determining the sources of resistance at seedling stage by comparing the response of the tested sources to the reaction of the isogenic lines allows investigating a large number of sources and the obtained information can be used for the development of resistant cultivars. The gene postulation, determined through the multipathotype test, is the most widely applied method worldwide for proving the presence of race specificity and for identification of certain Lr genes in different wheat populations (Statler, 1984; Modawi et al., 1985; Singh, Gupta, 1991; Singh, 1993; Singh et al., 1999; Oelke, Kolmer, 2004; Gebrewahid et al., 2017; Yan et al., 2017; Zhang et al., 2019; Wu et al., 2020). The multipathotype test, however, has certain shortcomings. Kadkhodaei et al. (2012) pointed out that the identification of Lr genes is rather labor and time consuming. Furthermore, there may be no available pathotypes suitable for identification of the genes for resistance present in the genotypes, or the pathotype may not be able to detect the genes for resistance to rust.

Starting from these premises and estimating the difficulties and disadvantages of the use of the multipathotype test, our investigation, too, could not achieve complete and thorough identification of a gene, but only a suggestion; on the basis of the response of these lines at seedling and adult stages, however, the nature of the resistance was determined, which also provides valuable data that can aid the breeding and improvement work for development of cultivars resistant to the disease.

The aim of this investigation was to study the response of common winter wheat lines both at seeding and adult stages and to use the obtained data on stable resistance present in these lines to aid the breeding for development of cultivars resistant to Puccinia triticina

## Materials and methods

In the infection field of Dobrudzha Agricultural Institute – General Toshevo, Bulgaria, the reaction to leaf rust (P. triticina) of 250 lines of common winter wheat involved in a competitive varietal trial was studied. From the investigated breeding material, 20 lines were selected, which responded with a certain degree of resistance from moderate to high (MR–VR), and which demonstrated resistant reaction to some of the used pathotypes of P. triticina at seedling stage under greenhouse conditions

Seedling test. The selected 20 lines were tested for resistance to single pathotypes of P. triticina and their response was compared to the reaction of a set of 34 differential lines (isogenic lines developed on the basis of cultivar Tatcher and each carrying one of the already identified Lr genes) according to the 7 pathotypes used in the study, which possessed different virulence (13763, 33762, 43773, 53723, 53762, 73762, and 73763). The pathotypes used in the test were identified on the basis of the reaction of 15 monogenic lines, Lr1, Lr2a, Lr2b, Lr2c, Lr3, Lr9, Lr11, Lr15, Lr17, Lr19, Lr21, Lr23, Lr24, Lr26 and Lr28, coded for by the method of Limpert and Müller (1994). Avirulence/virulence profiles of P. triticina pathotypes are present in Table 1.

**Table 1.**
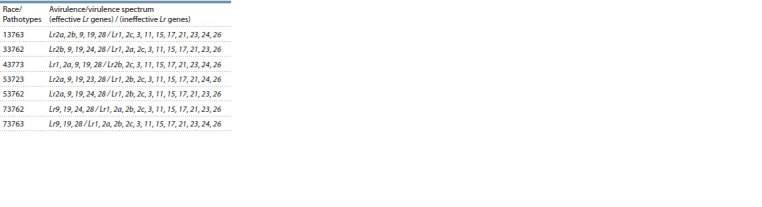
Avirulence/virulence profiles of Puccinia triticina pathotypes

The inoculated plants were placed in the dark in a moist chamber at temperature 18–20 °С and 100 % relative air humidity. After 24 hours at these conditions, they were transferred to a greenhouse for further growing under controlled conditions: 20–25 °C (day) and 15 °C (night), more than 75 % relative air humidity and 30,000 lx light intensity, for elongation of the photo period – 16 h (day) and 8 h (night)

In order to improve sporulation, the plants were treated with maleic hydrazide 97 % solution (1 g in 3 l water). On the 9–12th day after inoculation, the type of reaction (R) was read according to the scale of Stakman et al. (1962).

Infection types 0, 0;, 1 and 2 were considered expression of a resistant type of reaction (R), while infection types 3, 4 and X were considered susceptible (S) while estimating the disease.

Adult plant test. The investigation was carried out under conditions of a maximum infection created in the field, where the full set of pathotypes identified for the respective year were taken out. The lines were planted manually in 1.5 m wide rows with 0.25 m interspacing, in two replications. Cultivar Michigan amber was used as a multiplier and distributor of leaf rust. Spreader rows of M. amber were planted perpendicular and adjacent to the test rows. The artificial inoculation with the pathogen was done according to the methodology for working with rusts adopted at the Plant Pathology Laboratory of Dobrudzha Agricultural Institute (Ivanova, 2012). Nine-day old seedlings from the standard susceptible cultivar M. amber inoculated with different pathotypes of P. triticina were planted in the rows of the spreader cultivar in March and April till the final accumulation of inoculum in June, when the maximum was reached. The type of infection and the attacking rate were read according to the scale of Cobb, modified by Peterson (Peterson et al., 1948) at milk maturity stage. The average coefficient of infection (ACI), or the so called corrected relative attack rate (P0), was calculated by introducing a coefficient for the respective infection types (R – 0.2; MR – 0.4; M – 0.6; MS – 0.8; S – 1). Depending on the values of ACI, the studied lines were divided into several groups: immune (ACI = 0); very resistant, VR (ACI = 0–5.99); resistant, R (ACI = 6–25.99); moderately resistant, MR (ACI = 26–45.99); moderately susceptible, MS (ACI = 46–65.99); susceptible, S (ACI = 66–100). The lines with susceptible reaction were of no interest to us.

## Results and discussion

The experiment was carried out in three successive vegetative growth seasons. Out of the investigated 250 common winter wheat lines, 20 lines were selected, which responded with high to moderate resistance over the years of study. The lines responding with MR probably carry slow-rusting genes. According to Morgunov et al. (2010), some genes with slowrusting effect have a moderately susceptible type of infection but their attack rate does not exceed 50 %. The response of the lines investigated under field conditions is presented in Table 2, and the reaction of the lines at seedling stage to seven separate P. triticina pathotypes of different virulence is given in Table 3. The results of the investigation revealed the following.

**Table 2.**
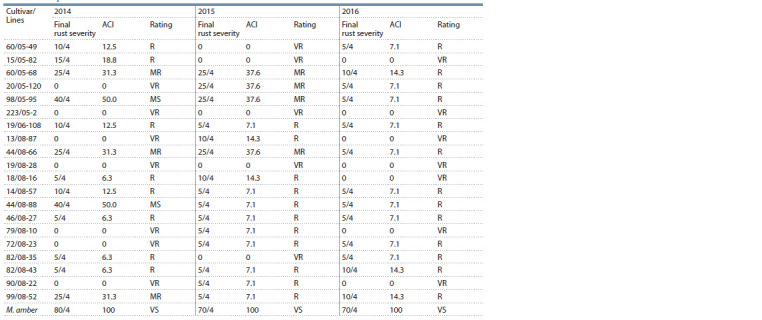
Adult plant resistance

**Table 3.**
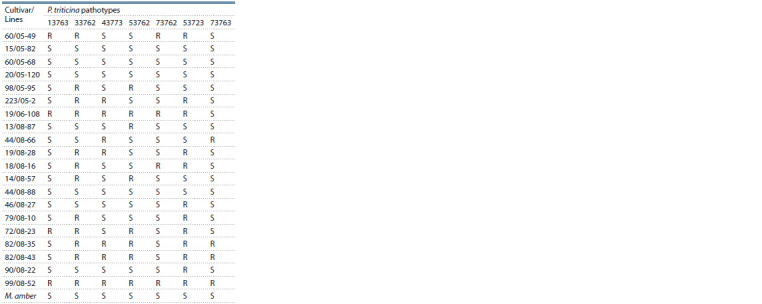
Response of common winter wheat lines to 7 pathotypes of P. triticina at seedling stage

Line 60/05-49 at seedling stage exhibited a resistant reaction to four phenotypically different pathotypes (see Table 3), and the field evaluation showed that this line had a resistant to very resistant reaction (see Table 2). This allows us to comment that the line is a carrier of partial race-specific resistance in combination with resistance of non-specific nature, but the race-specific resistance has to be checked at a later stage.

Line 15/05-82 demonstrated a susceptible reaction to all pathotypes of the pathogen used under greenhouse conditions, and the field evaluation showed that the line responded with a very resistant to resistant reaction. According to this reaction exhibited at seedling and adult stages, the line can be defined as a carrier of adult or field resistance

Line 60/05-68, also at seedling stage, responded with a susceptible reaction to all pathotypes used in the study, and the field evaluation showed a resistant to moderately resistant reaction. The response of the line allowed referring it to the group of the carriers of the slow rusting type of resistance

Line 20/05-120 at seedling stage responded with a susceptible reaction to all used pathotypes, and in the field it exhibited resistance of the type (VR–R–MR), but judging by the reaction, this line can be referred to the group of lines carrying resistance of race non-specific nature.

Line 98/05-95 at seedling stage demonstrated a resistant reaction to two pathotypes (33762 and 53762), and the field evaluation was not constant; in 2014, when the attack on cultivar M. amber was even higher in comparison to the other two years, the lines responded as moderately susceptible. In 2015 and 2016, the line demonstrated a resistant to moderately resistant reaction. This line carried resistance of race nonspecific nature

Line 223/05-2 responded with a resistant reaction to three pathotypes under greenhouse conditions and with complete resistance at adult stage. The line was a carrier of partial racespecific resistance in combination with race non-specific one

Line 13/08-87 at seedling stage responded with a resistant reaction to only one pathotype (53762), and its field resistance was of the VR–R type. The line was a carrier of resistance of race non-specific nature.

Line 44/08-66 responded with a resistant reaction at seedling stage to two pathotypes (43773 and 73763), and during two of the years it demonstrated field resistance of the MR type. Based on the response of the line, it was referred to the group of lines of the slow rusting type.

Line 18/08-16 as well as line 19/08-28 responded with a resistant reaction at seedling stage to three of the pathotypes and demonstrated R to VR under field conditions, allowing us to refer them to the group of lines combining partial racespecific resistance with race non-specific one. The combination of these two types of resistance in a single genotype is a good solution for breeding since the host is protected against diseases during the entire vegetative growth season.

Line 14/08-57 demonstrated stable field resistance and a resistant reaction to two of the pathotypes under greenhouse conditions. The line was a carrier of race non-specific resistance.

Line 46/08-27 reacted with stable resistance in the field during the three years of testing, and at seedling stage, it demonstrated a resistant reaction to only one pathotype. The line carries resistance of race non-specific nature.

Line 79/08-10 responded at seedling stage with a resistant reaction to two pathotypes (33762 and 53723), and in the field it demonstrated a resistant to very resistant reaction. The line is a carrier of race non-specific resistance.

Line 72/08-23 showed a resistant to very resistant reaction in the field, and under greenhouse conditions, a resistant reaction to four pathotypes was registered. The line is a carrier of partial race-specific resistance in combination with race non-specific one.

Line 90/08-22 exhibited a resistant to very resistant reaction in the field, while responding with a resistant reaction to only one pathotype at seedling stage. This line is probably a carrier of adult race specific resistance or resistance of race non-specific nature

Line 99/08-52 responded with a resistant reaction to all 7 pathotypes used in this study, and the field evaluation was within the range of R–MR. The presence of full resistance at seedling stage was a proof that the line possessed race-specific resistance. Its reaction coincided entirely with the reaction of the isogenic lines carrying genes Lr9, Lr19, Lr22a, Lr22b and Lr25 (Table 4).

**Table 4.**
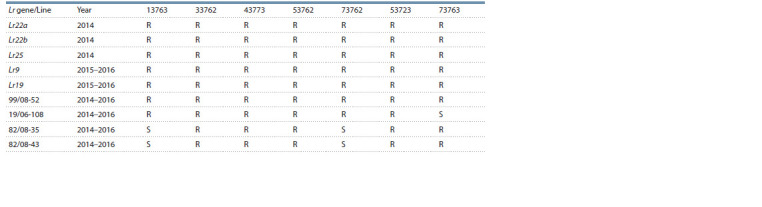
Comparative reaction between four breeding lines and the isogenic lines carrying genes Lr9, Lr19, Lr22a, Lr22b and Lr25, determining resistance to pathotypes of leaf rust

## Conclusion

As a result of this study, we identified the following types of resistance in the investigated lines:

Lines with race-specific resistance, which are to be subjected to breeding and genetic studies to prove the presence of the race-specific gene – four of the lines probably carried this type of resistance: 19/06-108, 99/08-52, 82/08-35 and 82/08-43. They constituted 20 % of all investigated lines

• Lines combining partial race-specific resistance at seedling stage with resistance of race non-specific nature at adult stage. The combination of race-specific with race nonspecific resistance is a good possibility to protect the host against the disease during the entire vegetative growth. The lines that fall in this group are 60/05-49, 223/05-2, 19/08-28, 18/08-16, 72/08-23. They constituted 25 % of the investigated lines.

• Lines-carriers of race non-specific resistance. The nonspecific nature of resistance is determined by the fact that at adult age, the host is resistant to all races and in this case the resistance is determined by 4 or 5 small genes with additive effect. Lines 15/05-82, 98/05-95, 14/08-57, 46/08- 27, 79/08-10, 90/08-22, 44/08-88 and 13/08-87 fell in this group. They constituted 40 % of all studied lines.

• Lines-carriers of the slow rusting type of resistance: lines 20/05-120, 44/08-66 and 60/05-68 belonged to this group and they constituted 15 % of the investigated material. The partial resistance is more durable than the resistance conditioned by single main genes since it is inherited polygenically (Parlevliet, 1985).

The lines studied in this investigation are carriers of certain types of resistance. According to Volkova et al. (2020), the cultivars with race-specific resistance are applied as a mosaic of varieties with subsequent alternation over time and space, and the cultivars that carry non-specific resistance can be used on large areas for a longer period of time in combination with cultivars from different groups, including their own.

In this relation, the studied lines carrying race-specific or race non-specific resistance can be included in the breeding programs for developing resistant cultivars in order to avoid large yield losses caused by the disease.

## Conflict of interest

The authors declare no conflict of interest.
